# Transporter-Mediated Solutes Uptake as Drug Target in *Plasmodium falciparum*

**DOI:** 10.3389/fphar.2022.845841

**Published:** 2022-02-07

**Authors:** Júlio César Monteiro Júnior, Arne Krüger, Giuseppe Palmisano, Carsten Wrenger

**Affiliations:** ^1^ Unit for Drug Discovery, Department of Parasitology, Institute of Biomedical Sciences, University of São Paulo, São Paulo, Brazil; ^2^ GlycoProteomics Laboratory, Department of Parasitology, Institute of Biomedical Sciences, University of São Paulo, São Paulo, Brazil

**Keywords:** malaria, transporters, new drugs, solute uptake, resistance, *P. falciparum*

## Abstract

Malaria remains a public health problem with still more than half a million deaths annually. Despite ongoing efforts of many countries, malaria elimination has been difficult due to emerging resistances against most traditional drugs, including artemisinin compounds - the most potent antimalarials currently available. Therefore, the discovery and development of new drugs with novel mechanisms of action to circumvent resistances is urgently needed. In this sense, one of the most promising areas is the exploration of transport proteins. Transporters mediate solute uptake for intracellular parasite proliferation and survival. Targeting transporters can exploit these processes to eliminate the parasite. Here, we focus on transporters of the *Plasmodium falciparum*-infected red blood cell studied as potential biological targets and discuss published drugs directed at them.

## Transporters in *Plasmodium falciparum*-Infected RBCs

The intracellular protozoan parasite *Plasmodium falciparum* causes the most severe form of malaria responsible for the majority of cases and deaths globally (Garcia 2010; World Health Organization 2021). When invading red blood cells during the asexual phase of the life cycle, the parasites hide themselves inside the so called parasitophorous vacuole (PV), which consists of the host cell membrane (Geoghegan et al., 2021). The survival and development of the intracellular parasite depend on the remodelling of the infected red blood cell (iRBC) (Desai 2014; Gilson et al., 2017). Major modifications include the biosynthesis, trafficking, and post-translational modification of transport proteins at different levels of the cell, such as the erythrocyte plasma membrane (EPM), the PV membrane (PVM) and the parasite plasma membrane (PPM) (Desai 2014; Basore et al., 2015; Pain et al., 2016; Counihan et al., 2021). The entire genomic complement of these transporters is termed the transportome with several subsets expressed at a given time and site (Martin 2020). The transportome is dynamic and differs substantially throughout the parasite’s life cycle which is subject of drug development studies (Martin et al., 2005; Counihan et al., 2021).

Early investigations into the *P. falciparum* transportome did not find high similarity with other eukaryotes. Less than 10% of the proteins of the five major eukaryotic transporter families (major facilitator superfamily (MFS), ATP-binding cassette (ABC) family, P-type ATPase family and the amino acid/polyamine/choline (APC) family) were found in *P. falciparum*, which suggested a restricted parasite-specific set of proteins specialized in transport (Gardner et al., 2002). However, with the advent of bioinformatics, the amount of identified transporters has expanded, encompassing more than 144 genes corresponding to 2.52% of the *Plasmodium* genome (Martin 2020). Nevertheless, it is a smaller number than found in other eukaryotes, such as *Saccharomyces cerevisiae* (5.4%) and *Homo sapiens* (4.3%) (Ren et al., 2007). Gene knockouts have been widely used to assess which transporters are essential for parasite survival (El Bissati et al., 2008; Slavic et al., 2010; Staines et al., 2010; Summers and Martin 2010; Ito et al., 2017). These studies suggest absence of functional redundancy (exceptions mentioned below) in the transportome, which means transporters primarily are substrate-specific and not permeable to multiple molecules (Martin et al., 2005). Additionally, about 2/3 of these genes were reported to be essential for parasite proliferation in the intraerythrocytic phase (Martin 2020). Minor homology to the human transportome and major essentiality for the parasite qualify the parasite-specific transporters as potential new drug targets (Staines et al., 2010; Gupta et al., 2015).

Transport proteins can be categorized into ion channels, carriers, and pumps. Ion channels are water-filled pathways that allow passage of ions through the lipid membrane and are often gated by different stimuli, such as ligand binding or voltage change (Staines et al., 2010; Martin 2020). They allow fast diffusion of solutes down their transmembrane electrochemical gradients (Martin 2020). The passage depends on solute affinity for an internal site of the channel (Gezelle et al., 2021). Carriers, on the other hand, undergo a conformational change to allow passage of a specific solute (Desai 2014). If the transport is down the electrochemical gradient, the carrier is called an uniporter (Martin 2020). However, if the transport is against the electrochemical gradient, the carriers can use the potential difference (secondary source energy) coming from the unbalance of ion concentrations. The most common are Na^+^, K^+^ and H^+^, which are used as cotransporters, either in the same direction as the main substrate (symporters), or in the opposite (antiporters) (Staines et al., 2010; Martin 2020). Pumps are a type of carrier that use a primary source of energy (e.g., ATP) for solute transport against the electrochemical gradient. Due to this dynamic change, carriers and pumps have the lowest transport rate (Gezelle et al., 2021). In this review, we will focus on the druggability of those transporters including their respective compounds, since transporter biology and nutrient acquisition of *Plasmodium* spp. have been subject to recent comprehensive reviews (Martin 2020; Beck and Ho, 2021; Counihan et al., 2021).

### Challenges in Transporter Drug Screening

In antiplasmodial drug development three factors are of concern: 1) essentiality, that describes if the target is essential for parasite survival; 2) toxicity, that indicates potential side effects on human orthologues, and 3) druggability, that determines if a drug can be developed specifically for the target (Staines et al., 2010; Meier et al., 2018).

Applying knock-out studies to the haploid asexual *Plasmodium* spp. genome can identify essential transporters as drug targets (El Bissati et al., 2008; Slavic et al., 2010; Staines et al., 2010; Summers and Martin 2010; Ito et al., 2017). When an essential transporter is identified, compounds can be tested using either whole-cell or *in vitro* screenings. The whole-cell system allows a more integrated view of the compound’s action on the complex biology of the parasite but identifying the individual components of the transport process and characterizing the drug-target interaction and mode of action is challenging (Plouffe et al., 2008; Chatterjee and Yeung, 2012; Moffat et al., 2017). Isolating the compound’s effect on a specific transporter is almost impossible due to the presence of other types of transporters and potential downstream effects (Martin 2020). At the same time it impedes drug optimization and reduction of toxicity (Chatterjee and Yeung, 2012; Penzo et al., 2019). The complexity of the plasmodial endomembrane system further complicates target-specific analyses (Martin 2020). Attempts to circumvent these issues include overexpression of the transporter in the parasite or expression in a heterologous systems, such as yeast (Frame et al., 2015; Sosa et al., 2019), but both techniques have drawbacks (Martin 2020). Target-based screens rarely yield a good drug (Plouffe et al., 2008) because drug-target interaction depends on physiochemical parameters (e.g., solubility, permeability) which can be better evaluated in a whole-cell system (Chatterjee and Yeung, 2012). Additionally, some *Plasmodium* organelles or membrane structures (e.g., DV) are simply not present in other organisms hindering the heterologous assessment. Nonetheless, transporter-inhibitor characterizations can be accomplished employing sophisticated techniques that require expertise and cost, such as the patch-clamp method [for a review and guide, see Gezelle et al. (2021)]. This methodology allows the electrophysiological characterization of transporters, both in the whole-cell and in the single-channel system, under varying conditions.

Another important factor related to druggability is the stage-dependent effect of the inhibitor (Whitehead and Peto 1990; Rottmann et al., 2010; Jiménez-Díaz et al., 2014). Transporter expression varies immensely during the asexual blood-stage which is why it is fundamental to understand not only the localization but also the time the transporter is present. This will increase the effect and reduces the risk of toxicity (Baruah et al., 2017). Armed with these information and techniques it has been possible to assess the potential of several transporters as new drug targets as discussed in the following.

### The Role of Erythrocyte Plasma Membrane Transporters in Solute Uptake

The survival of the parasite inside the erythrocyte depends on the access to nutrient molecules from the extracellular space. Access is achieved by trafficking proteins from the parasite to the EPM to adjust permeability to solutes (Desai 2014). Targeting transporters in the EPM with drugs leaves few options for the parasite to develop resistance, which is normally mediated by transporters that facilitate the efflux of drugs such as the *P. falciparum* chloroquine resistance transporter (*Pf*CRT) and *P. falciparum* multidrug resistance protein 1 (*Pf*MDR1) (Staines et al., 2010; Haldar et al., 2018). Possible mechanisms are limited to mutations in the targeted transporter itself modifying the affinity to the drug which at the same time could harm the permeability to important solutes.

**FIGURE 1:**
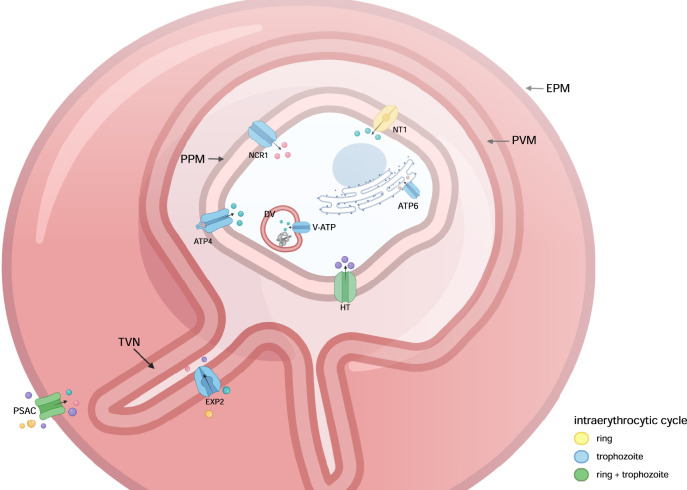
In the *P. falciparum*-infected red blood cell, several transporters represent potential drug targets. The only *Plasmodium* spp. transporter in the EPM so far being evaluated as a drug target is PSAC responsible for passage of a broad range of solutes. Not too many transporters are known at the PVM with EXP2 being suggested as a potential target recently, forming a non-specific high-conductivity pore. The PPM contains most transporters investigated as drug targets, such as HT, FNT, NT1, NCR1 and ATP4. Inside the parasite, transporters facilitate function of diverse organelles, such as ATP6 in the endoplasmic reticulum. The color scheme of transporters indicates in which stages of the intra-erythrocytic life cycle the is expressed. EMP, erythrocyte plasma membrane; PVM, parasitophorous vacuolar membrane; TVN, tubovesicular network; PPM, parasite plasma membrane; PSAC, plasmodial surface anion channel; VDAC, voltage-dependent anion channel; EXP2, exported protein 2; HT, hexose transporter; NT1, nucleoside transporter 1; NCR1, Niemann-Pick type C1-related protein; ATP4, P-type Na^+^-ATPase 4; ATP6, sarco/endoplasmic reticulum Ca^2+^-ATPase; DV, digestive vacuole.

One major contributor to solute permeability is the *clag* multigene family with the products from paralogs *clag3.1* and *clag3.2* being related to the formation of the plasmodial surface anion channel (PSAC) (Figure 1) (Nguitragool et al., 2011; S. A.; Desai et al., 2000). PSACs are externalized to the EPM about 20 h post infection (hpi) at the trophozoite stage and remain throughout the cycle, representing the main route of solute uptake for a broad range of solutes including monosaccharides, purines, pantothenate (coenzyme-A precursor), and amino acids isoleucine and methionine (Gupta et al., 2015; Martin 2020). Further, *clag3* switching achieved by the monoallelic expression of the paralogs allows for a fine-tuning of the channel with subtle modifications to cover the affinity to the different solutes which could be shown via the aforementioned patch-clamp method (Nguitrangool et al., 2012; Gupta et al., 2015; Gupta et al., 2018). This points to an important pathway for the parasite nutrition in expressive quantity and diversity of substrates, including drug uptake, such as blasticidin S and leupeptin (Lisk et al., 2010). PSACs are composed of parasite proteins without any known human orthologue, a desirable feat in drug development.

First screens against PSAC were conducted with known drugs, such as furosemide derivatives (Table 1). Furosemide caused a delay in protein biosynthesis at high concentrations which could be ascribed to blocked isoleucine influx via PSAC/NPP (Martin and Kirk 2007). Isoleucine uptake is essential for the development of the parasite, since it is absent from human hemoglobin. Its transport is characterized as the antiport of isoleucine and leucine in a saturable process (within normal physiologic range) and is independent of ATP or Na^+^ and H^+^ (Martin and Kirk 2007). However, there is not much information about inhibitors acting directly on the isoleucine uptake over the PPM.

**TABLE 1:** Overview of *Plasmodium*-infected RBC transporters tested as novel antimalarial drug targets.

Target	Localization	Drug	References
**PSAC**	EPM	PRT, ISPA-28, furosemide derivatives	Staines et al. (2010); Nguitragool et al. (2011); Pain et al. (2016)
***Pf*ATP4**	PPM	(+)-SJ733, MB14, spiroindolones, cyclopiazonic acid	Krishna et al. (2001); Rottmann et al. (2010); Spillman and Kirk (2015); Gilson et al. (2019)
***Pf*HT**	PPM	cytochalasin B, TCMDC125163, C3361, lopinavir	Woodrow, et al. (2000); Joët et al. (2003); Slavic et al. (2010); Ortiz et al. (2015); Jiang et al. (2020); Kraft et al. (2015); van Niekerk et al. (2016)
***Pf*NT1**	PPM	ChemBrigde ID 9001893, ChemBrigde ID 6946484, furamide and benzamide derivatives	Carter et al. (2000); Parker et al. (2000); Frame et al. (2015)
***Pf*NCR1**	PPM, DV	MMV009108, MMV019662, MMV028038	Istvan et al. (2019)
**(V/P)-type-ATPase**	EPM/PPM/PVM	bafilomycin A1, concanamycin B	Hayashi et al. (2000); Marchesini et al. (2000); Tang et al. (2019)
***Pf*ATP6**	ER	Atelorane, thaspsigargin	Crespo et al. (2008); Abiodun et al. (2013)

Newer drugs specifically targeting PSAC such as ISPA-28 (Table 1) have been very promising with K_0.5_ values of 56 nM and 43 μM for *P. falciparum* strains Dd2 (*clag3.1* expression) and HB3 (*clag3.2* expression), respectively. This difference in action is due to the *clag3* switching in PSAC formation. Only CLAG3.1 contains an extracellular, hypervariable region (HVR). ISPA-28 interacts with a specific polymorphism in the HVR only present in the Dd2 CLAG3.1 (Nguitragool et al., 2011; Nguitragool et al., 2014; Gupta et al., 2018; Mira-Martínez et al., 2019; Gupta et al., 2020). Although promising, the distinct efficacy depending on expression switching and presence of an HVR pose a challenge for PSAC as a novel antimalarial drug target.

In contrast to the parasite-derived PSAC, host cell transporter such as the erythrocyte voltage dependent anion channel (VDAC) could be potential targets (Bouyer et al., 2011; Gezelle et al., 2021). VDACs may compose the peripheral-type benzodiazepine receptor (PBR), known and widely used as target for other drugs such as neuromodulators (Bouyer et al., 2011). Therefore, some studies have also tested PBR ligands against malaria to reduce the VDACs conductivity. The PBR antagonist isoquinoline carboxamide (PK11195) showed a comparably high IC_50_ value of 10 µM against parasites in culture (Bouyer et al., 2011). This study focused on the physiology of the transporter and taken together with the high risk for toxicity of targeting a host cell transporter might explain why little follow-up studies against VDACs were conducted (Staines et al., 2010).

Before reaching the PPM, all solutes need to pass the PVM surrounding the parasite (Figure 1). Formerly, presence of a non-selective channel was thought to allow the free passage of solutes (Desai 2014; Spillman and Kirk, 2015). Recently, a large and permeable pore was suggested to allow the passage of solutes <1.3 kDa (Mesén-Ramírez et al., 2021 (Beck and Ho, 2021); formed by exported protein 2 (EXP2) (Mesén-Ramírez et al., 2019). However, the biology of the PVM and its channels in the physiology of the parasite is still poorly understood although the ion concentration within the PV is favorable for the function of transporters in the PPM (discussed below). While most parasite-derived transporters are highly solute-specific, PSACs and the PVM pore are permeable to a broad range of solutes, underlining their importance for the physiology of the parasite.

### The Role of Parasite Plasma Membrane Transporters in Solute Uptake

Solutes need to ultimately pass the PPM via a diverse set of transporters to be accessible to the parasite (Desai 2012). Lipid transport mediated by the *P. falciparum* Niemann-Pick type C1-related proteins (*Pf*NCR1) (Figure 1) is required for the formation of the endomembrane system, as parasites with malfunction of these transporters have a fragile PPM more susceptible to lysis (Beck and Ho, 2021). Lipids transported via *Pf*NCR1 are further important for digestive vacuole (DV) membrane formation. The DV is the digestion site for hemoglobin, the main amino acid source used by the parasite. Hemoglobin digestion occurs mainly during the trophozoite-stage (Milani et al., 2015). Thus, *Pf*NCR1 inhibition may compromise the integrity of the DV, impairing hemoglobin metabolism (Istvan et al., 2019). Compounds MMV009108 and MMV019662 (Table 1) from the malaria box were tested against *Pf*NCR1 in culture including resistance selection assay. Both compound inhibited parasite growth with an IC_50_ of ∼500 nM but lost effect due to resistance selection over time caused by mutations in the transporter itself (Istvan et al., 2019).

The *P. falciparum* hexose transporter (*Pf*HT) (Figure 1) is an essential protein that transports hexoses (e.g. glucose) over the PPM for the parasite’s anaerobic glycolytic metabolism (Jiang et al., 2020). *Pf*HT expression peaks in the early-ring stage decaying throughout the intraerythrocytic cycle (Woodrow et al., 1999). The human glucose transporter (GLUT1) is structurally different from *Pf*HT reducing the chance of possible side effects. Compound TCMDC-125163 (Table 1) from the TCAMS library exhibited an IC_50_ = 39 nM against *Pf*HT versus 3.2 µM for GLUT1 (Ortiz et al., 2015). Recently, the interaction between *Pf*HT and a small-molecule glucose derivative (C3361) was demonstrated through co-crystallization. C3361 binds to *Pf*HT inducing a structural rearrangement preventing glucose passage and culminating in the formation of an additional pocket, which can be exploited to enhance carrier inhibition (Jiang et al., 2020). The IC_50_ of C3361 was determined as 30 μM for *Pf*HT and 1.3 mM for GLUT1 proving selectivity for the parasite transporter (Jiang et al., 2020). Lopinavir (Table 1), an HIV protease inhibitor, is known for its antimalarial activity (IC_50_ = 1.9 µM against *P. falciparum* 3D7 culture) although the target remained obscure. Kraft and colleagues identified *Pf*HT as the antiviral’s target acting as a competitive inhibitor of glucose uptake by binding to a single pocket on the intracellular side, preventing glucose transport. However, inhibition was not as potent as with C3361 and full effects of lopanivir on GLUT isoforms are not yet known (Kraft et al., 2015). Studies on *Pf*HT are promising and point to it as one of the strong targets for novel antimalarial drug design.

*Plasmodium* parasites lack a purine biosynthesis pathway, thus depend on their acquisition from the host. The *P. falciparum* nucleoside transporter (*Pf*NT1) (Figure 1) was shown to mediate not only purine but also pyrimidine nucleoside uptake (Carter et al., 2000; Staines et al., 2010). *Pf*NT1 is primarily expressed during the early blood stages (Carter et al., 2000). In initial studies, furamide and benzamide derivatives (Table 1) inhibited recombinantly expressed *Pf*NT1 with IC_50s_ < 50 µM (Frame et al., 2015). A recent high-throughput screening of GlaxoSmithKline’s drug library applying a special growth assay with *Pf*NT1-expressing yeast identified six hits. The IC_50_ values of *Pf*NT1 inhibitors were similar (<20 µM) for a variety of resistant and non-resistant *P. falciparum* strains subsequently tested, proving a distinct mode of action against *Pf*NT1 (Sosa et al., 2019).

One of the modifications in the intracellular environment for the proper development of the parasite is the increase of Na^+^ in iRBC cytosol, while the parasite maintains a low Na^+^ concentration, generating an important electrochemical gradient for nutrient transport. This imbalance is achieved through three transporters: 1) new permeability pathways (NPPs) in the EPM that are freely permeable to Na^+^; 2) pores in the PVM which, in the trophozoite stage, are largely free for the passage of solutes (Spillman and Kirk, 2015); and 3) pumps such as *P falciparum* P-type Na^+^-ATPase (*Pf*ATP4) (Figure 1) responsible for the efflux of Na^+^ over the PPM while importing H^+^ (Spillman and Kirk, 2015). Spiroindolones such as NITD609 (rebranded as cipargamin) (Table 1) act by blocking the antiport of Na^+^ and protons, leading to an altered electrochemical gradient and alkalinization of the parasite, respectively (Spillman and Kirk, 2015). NITD609 was initially shown to inhibit protein biosynthesis in the parasite but resistance mechanisms were linked to mutations in *pfatp4* (Rottmann et al., 2010). Follow-up studies revealed inhibition of *Pf*ATP4 and suggested it as the primary target of the spiroindolones (Spillman et al., 2013). *In silico* docking showed that the NITD609-*Pf*ATP4 interaction is driven by nonpolar residues. Substituting the interacting amino acids with polar residues (L290S and P339T) impaired binding affinity (Goldgof et al., 2016). Tests of NITD609 in culture on several field isolates of *P. falciparum* and *P. vivax*, including drug resistant and susceptible isolates, resulted in the same inhibitory potential in the low nanomolar range (Rottmann et al., 2010). The drug further inhibits gametocyte and oocyst development, acts faster than artemisinins, could cure *in vivo* infections with *P. berghei* in a single dose (100 mg/kg), and possesses favorable pharmacokinetic and pharmacodynamic properties (Rottmann et al., 2010; Dick et al., 2020). NITD609 already concluded the first phase 2 clinical trial with another phase 2 trial planned for March 14, 2022 (Novartis Pharmaceuticals 2022); accessed 18/02/2022).

A second drug against *Pf*ATP4 to enter clinical trials is the dihydroisoquinolone (+)-SJ733 (Table 1) which specifically induces senescence in iRBCs. The interaction between *Pf*ATP4 and (+)-SJ733 occurs at the kink in a transmembrane alpha helix (residues 406-410) of *Pf*ATP4, the same pocket used as NITD609 binding site (Goldgof et al., 2016). The surrounding region contains resistance residues, in addition to a loop with high variability between *Plasmodium* spp. which can lead to varying sensitivity to (+)-SJ733 (Jiménez-Díaz et al., 2014). However, when tested, the variation in efficacy of (+)-SJ733 did not change significantly between strains such as 609, 3D7, K1 and D2, or at different stages of the intraerythrocytic cycle (Jiménez-Díaz et al., 2014). Despite a rapid parasite clearance time of 3.56 h (95% CI 3.29–3.88 h) for 600 mg in clinical trials (+)-SJ733 effect is not sustained, and recrudescence occurs approximately 60 h after treatment. Therefore (+)-SJ733 needs an association with a slow-acting drug to improve its effect or a periodical multidose approach (study ongoing). *Pf*ATP4 is the first novel drug target in *Plasmodium* spp. to be clinically validated since the 1980s (Gaur et al., 2020).

### The Role of Organelle Transporters

Organelles such as the DV, mitochondria and endoplasmic reticulum are also dependent on transport processes over their membrane for proper function. The activity of ATPases depends on the aforementioned imbalance of ions between PV and parasite (secondary source energy) (Spillman and Kirk, 2015). (*Pf*ATP6) is a calcium pump located at the endoplasmic reticulum and responsible for maintaining the parasite’s calcium balance. Originally, it was thought to be a target of artemisinin and involved in resistance (Eckstein-Ludwig et al., 2003; Adhin et al., 2012). However, later studies could prove these findings wrong (Arnou et al., 2011; Cardi et al., 2010). Sensitivity tests of thapsigargin (Table 1) against *Pf*ATP6 revealed IC_50_s around 4 µM in *P. falciparum* strain 3D7 (Crespo et al., 2008). However, *Pf*ATP6 does not qualify as the best drug target since it is an ortholog of human sarco/endoplasmic reticulum Ca^+2^-ATPase (SERCA) (Eckstein-Ludwig et al., 2003; Adhin et al., 2012). Another way to target DV is using bafilomycin A1 (Baf-A1) (Table 1) (Saliba and Kirk 1999; Marchesini et al., 2000). This drug belongs to a family of macrocyclic lactones that is tested as an inhibitor of vacuolar H^+^-ATPase (V-ATPase) (Figure 1) (Yatsushiro et al., 2005). These proton pumps are present throughout the parasite to ensure its acidic homeostasis. Acidification of the DV is important for the digestion of substrates (e.g., hemoglobin) (Yatsushiro et al., 2005; Tang et al., 2019). Baf-A1 acts by preventing acidification and consequently maturation of the DV, thus interrupting the parasite’s nutrition. Baf-A1 tested against 3D7 strain *in vitro* showed an IC_50_ value of 25 nM (Tang et al., 2019). Several other pumps shared by the host cell and parasite are exploited in the development of drugs against other diseases. The V/P-types ATPases are widely distributed in the parasite and are known to export ions coupled with Na^+^ or K^+^ (Dyer et al., 1996; Krishna et al., 2001; Staines et al., 2010; Tang et al., 2019). These pumps are already used in humans as drug targets against heart disease (Na^+^/K^+^ pumps) and gastropathy (H^+^/K^+^ pumps) (Staines et al., 2010).

Potential drug candidates with their respective targets are summarized in Table 1.

## Conclusion

The field of drug development against plasmodial transport proteins related to substrate uptake is just beginning. Targeting transporters has the potential to surpass the most concerning mechanisms of resistance identified so far. In this sense, understanding the molecular basis and physiology of solute uptake in various transporters can be a key strategy to combat malaria. Although the *P. falciparum* transportome is not entirely known, some transporters already are well characterized, and some drug candidates already entered clinical trials. However, there is still a lack of information about many possible targets and it remains challenging assess transporter activity. Therefore, it is necessary to expand information on the biology of malaria transporters, such as their structural characteristics, interaction partners, and their repertoire of substrates. Furthermore, it is essential to know the location of the transporters and in which phases of the life cycle they are present to better determine the treatment window. Therefore, future investigations are urgently required to better understand the transport processes in *Plasmodium* parasites and fuel transporter drug discovery.
